# Factors predicting adequate lymph node yield in patients undergoing pancreatoduodenectomy for malignancy

**DOI:** 10.1186/s12957-016-1005-3

**Published:** 2016-09-20

**Authors:** Marek Sierzega, Łukasz Bobrzyński, Andrzej Matyja, Jan Kulig

**Affiliations:** First Department of Surgery, Jagiellonian University Medical College, 40 Kopernika Street, 31-501 Krakow, Poland

**Keywords:** Cancer of the ampulla of Vater, Cancer staging, Distal common bile duct cancer, Lymph nodes, Pancreatic cancer

## Abstract

**Background:**

Most pancreatoduodenectomy resections do not meet the minimum of 12 lymph nodes recommended by the American Joint Committee on Cancer for accurate staging of periampullary malignancies. The purpose of this study was to investigate factors affecting the likelihood of adequate nodal yield in pancreatoduodenectomy specimens subject to routine pathological assessment.

**Methods:**

Six hundred sixty-two patients subject to pancreatoduodenectomy between 1990 and 2013 for pancreatic, ampullary, and common bile duct cancers were reviewed. Predictors of yielding at least 12 lymph nodes were evaluated with a logistic regression model, and a survival analysis was carried out to verify the prognostic implications of nodal counts.

**Results:**

The median number of evaluated nodes was 17 (interquartile range 11 to 25), and less than 12 lymph nodes were reported in surgical specimens of 179 (27 %) patients. Tumor diameter ≥20 mm (odds ratio [OR] 2.547, 95 % confidence interval [CI] 1.225 to 5.329, *P* = 0.013), lymph node metastases (OR 2.642, 95 % CI 1.378 to 5.061, *P* = 0.004), and radical lymphadenectomy (OR 5.566, 95 % CI 2.041 to 15.148, *P* = 0.01) were significant predictors of retrieving 12 or more lymph nodes. Lymph node counts did not influence the overall prognosis of the patients. However, a subgroup analysis carried out for individual cancer sites demonstrated that removing at least 12 lymph nodes is associated with better prognosis for pancreatic cancer.

**Conclusions:**

Few variables affect adequate nodal yield in pancreatoduodenectomy specimens subject to routine pathological assessment. Considering the ambiguities related to the only modifiable factor identified, appropriate pathology training should be considered to increase nodal yield rather than more aggressive lymphatic dissection.

## Background

Precise pathologic information is essential for clinical decision-making in patients with solid tumors, including those in the pancreaticoduodenal area (i.e., pancreatic, common bile duct, and ampullary cancers). Therefore, minimizing the risk of misclassification by harvesting an adequate number of lymph nodes is important not only for prognostic stratification but also for implementation of adjuvant therapy when indicated.

The accuracy of staging lymph node status is directly proportional to the number of lymph nodes retrieved and the optimum cutoff value minimizing the stage migration phenomenon reported previously for pancreatic cancer varies from 10 to 15 [1, 2]. Moreover, many studies suggested that removing at least ten lymph nodes is significantly associated with improved survival regardless of the presence of nodal metastases [1, 3, 4]. Others suggested that pathologic assessment of more than 12 lymph nodes may provide more accurate survival estimates for patients with node-negative disease [5, 6]. Based on these observations, at least 12 lymph nodes are required for adequate staging for pancreatoduodenectomy specimens of pancreatic, distal bile duct, and ampullary cancer according to the most recent edition of the American Joint Committee on Cancer (AJCC) TNM classification [7]. Contrary to these recommendations only about seven to eight nodes are dissected in many institutions worldwide [2, 3, 8–10]. This carries the risk of understaging, as an inadequate assessment of regional lymph nodes may erroneously identify node-positive patients as node negative.

Several previous reports demonstrated marked improvements in lymph node counts by adopting adequate methods of specimen processing by dedicated pathologists [11–14]. Surprisingly, there are hardly any studies that discuss other factors affecting retrieval of the optimal 12 lymph nodes according to the current recommendations of AJCC in patients undergoing pancreatoduodenectomy for cancers of the periampullary area. As understaging may have important therapeutic implications in routine clinical practice, the aim of this study was to investigate the impact of clinical and pathological factors on the likelihood of identifying the appropriate number of lymph nodes for cancers of the pancreatic head, ampulla of Vater, and common bile duct.

## Methods

### Patients

All patients undergoing pancreatic resections between 1990 and 2013 at our academic tertiary surgical center were reviewed to identify pancreatoduodenectomies carried out for malignancy of the pancreatic head, distal bile duct, and ampulla of Vater. Patients operated for non-malignant conditions were excluded. All data were prospectively collected and recorded in a dedicated database. Variables potentially affecting the number of lymph nodes identified in surgical specimens were retrieved from the database and analyzed retrospectively, including demographic data, pathologic features of the tumor, and therapeutic interventions. Follow-up data was collected based on clinical examinations performed every 3–6 months after discharge and dates of death from the census registry office. The study was approved by the Bioethics Committee of Jagiellonian University.

### Surgical procedures and pathological evaluation

All procedures were carried out by senior consultant surgeons experienced in pancreato-biliary surgery and using a similar technique of dissection. Primary tumors were resected en bloc with pancreaticoduodenal lymph nodes (groups 13 and 17 according to the Japanese Society of Biliary Surgery (JSBS) [15] and Japan Pancreas Society (JPS) [16]), whereas all other nodal stations were dissected separately. The extent of lymphadenectomy was described by the operating surgeon and classified as defined by the recent guidelines [17, 18]. Briefly, standard lymphadenectomy included resection of the following lymph node groups: anterior and posterior pancreaticoduodenal (nos. 13 and 17), hepatoduodenal ligament (no. 12), nodes to the right side of the superior mesenteric artery from its origin at the aorta to the inferior pancreaticoduodenal artery (nos. 14a and 14b), lymph nodes around the common hepatic artery (no. 8a), and celiac trunk (no. 9), suprapyloric (no. 5), and infrapyloric (no. 6) lymph nodes. Radical lymphadenectomy included removal of lymph node groups described for standard pancreatoduodenectomy along with para-aortic lymph nodes (nos. 16a2 and 16b1) located between the level of coeliac trunk and inferior mesenteric artery. The choice of surgical technique and the extent of lymphadenectomy was made at the discretion of the operating surgeon without any preoperative allocation. Lymph nodes were identified and retrieved from formalin-fixed surgical specimens by the pathologists without any specific techniques aimed at increasing nodal retrieval. In patients subject to total pancreatectomy, groups of lymph nodes located around the pancreatic body and tail (i.e., nos. 10, 11, and 18) were not included in nodal counts for the purpose of this study as they are not dissected at pancreatoduodenectomy.

### Statistical analysis

Mann-Whitney *U* and *χ*^2^ tests were used where appropriate to identify the significant factors predictive of retrieving at least 12 lymph nodes. Predictors significantly associated with nodal count were used for the development of a multivariate logistic regression model. The probability for entering the model was 0.05 and for removal from the model 0.100. Survival data was analyzed according to the Kaplan-Meier method and included postoperative mortality. The log-rank test was used to detect differences between groups. All tests were two-sided and *P* < 0.050 was considered statistically significant. Statistical analysis was performed using the IBM^®^ SPSS^®^ Statistics v.21 software package (IBM Corporation, NY).

## Results

### Study population

Among 842 pancreatoduodenectomies identified in our database between 1990 and 2013, 662 were carried out for pancreatic, ampullary, and common bile duct cancers. A group of 180 patients were excluded due to the final diagnosis of benign pancreatic disorders (*n* = 103) or other malignancies (*n* = 77). Clinical and demographic data of the selected population are summarized in Table 1.

**Table 1:** Baseline characteristics of the patients (*n* = 662)

Variable	
Gender (F:M)	263:399
Age, median (IQR) years	60 (50–66)
Final diagnosis, *n* (%)	
Pancreatic cancer	388 (59)
Cancer of the ampulla of Vater	236 (36)
Common bile duct cancer	38 (5)
Comorbidities, *n* (%)	
Cardiocirculary	252 (38)
Pulmonary	33 (5)
Diabetes	132 (20)
Cirrhosis	7 (1)
ASA class, *n* (%)	
I or II	470 (71)
III or IV	192 (29)
Preoperative biliary drainage, *n* (%)	
None	364 (55)
Endoscopic	179 (27)
Operative	119 (18)
Body mass index, median (IQR)	24 (21–26)
Surgery, *n* (%)	
Pancreatoduodenectomy (PD)	358 (54)
Pylorus-preserving PD	185 (28)
Total pancreatectomy	119 (18)

*ASA* American Society of Anesthesiologists, *IQR* interquartile range, *PD* pancreatoduodenectomy

### Evaluation of lymph nodes

During the study period, 444 (67 %) resection specimens were assessed onsite by one senior gastrointestinal pathologist (KN) and the remaining 218 (37 %) at a cooperating university pathology center. The median number of evaluated nodes was 17 (interquartile range 11 to 25, range 2 to 92), and less than 12 lymph nodes were reported in surgical specimens of 179 (27 %) patients. Table 2 shows detailed pattern of lymph node distribution. The highest median nodal yield was found for the pancreaticoduodenal lymph nodes (group nos. 13 and 17), followed by para-aortic (no. 16) and hepatoduodenal ligament nodes (no. 12). Overall, positive lymph nodes were identified in 396 (60 %) patients, including 264 patients with pancreatic cancer, 113 with ampullary cancer, and 19 with common bile duct cancer. There was no significant variability over time in the number of identified lymph nodes (correlation coefficient *r* = 0.086, *P* = 0.741) or the proportion of patients with 12 or more nodes examined (*r* = 0.124, *P* = 0.660). Median node counts were comparable among all operating surgeons and were not associated with the type of the primary tumor. There was a highly significant negative correlation between patients’ body mass index (BMI) and the proportion of patients with ≥12 lymph nodes examined (correlation coefficient *r* = –0.679, *P* = 0.002), but no such association was found for the absolute node count (correlation coefficient *r* = –0.056, *P* = 0.349).

**Table 2:** Pattern of lymph node distribution (*n* = 662)

Group according to JSBS/JPS	Location	Median (IQR) of examined nodes	No. (%) of patients with metastatic nodes by cancer site		
			Pancreatic *n* = 388	Ampullary *n* = 236	Bile duct *n* = 38
5	Gastric lesser curve and suprapyloric	2 (1–3)	5 (1)	2 (1)	0
6	Gastric greater curve and infrapyloric	3 (1–5)	12 (2)	2 (1)	0
8	Common hepatic artery	2 (1–3)	49 (13)	6 (3)	0
9	Celiac trunk	2 (1–4)	24 (6)	4 (2)	0
12	Hepatoduodenal ligament	3 (2–5)	57 (15)	13 (6)	2 (5)
13 and 17	Pancreaticoduodenal	8 (6–12)	238 (61)	104 (44)	19 (50)
14	Superior mesenteric artery	2 (1–3)	44 (11)	6 (3)	2 (5)
16	Para-aortic^a^	4 (2–6)	40 (10)	7 (4)	2 (5)
Overall	All stations	17 (11–25)	264 (68)	113 (48)	19 (50)

*Abbreviations*: *JSBS* Japanese Society of Biliary Surgery, *JPS* Japan Pancreas Society, *IQR* interquartile range

^a^Only patients with para-aortic lymph node dissection (*n* = 178)

### Predictive factors for lymph node yield

Table 3 shows results of a univariate analysis of factors associated with removal of at least 12 lymph nodes. Subsequent regression analysis, summarized in Table 4, identified only three independent predictors for adequate nodal yield, i.e., tumor diameter ≥20 mm (odds ratio [OR] 2.547, 95 % confidence interval [CI] 1.225 to 5.329), lymph node metastases (OR 2.642, 95 % CI 1.378 to 5.061), and radical lymphadenectomy (OR 5.566, 95 % CI 2.041 to 15.148).

**Table 3:** Univariate analysis of factors associated with removal of at least 12 lymph nodes

Factor	Lymph node count		*P* ^b^
	<12 (*n* = 179)	≥12 (*n* = 483)	
Gender			0.171
Female	79 (44)	184 (38)	
Male	100 (56)	299 (62)	
Age			0.125
<70 years	161 (90)	401 (83)	
≥70 years	18 (10)	82 (17)	
Cancer site			0.225
Pancreas	88 (49)	300 (62)	
Ampulla of Vater	79 (44)	157 (33)	
Common bile duct	12 (7)	26 (5)	
Comorbidities			0.893
No	90 (50)	237 (49)	
Yes	89 (50)	246 (51)	
ASA class			0.259
I or II	134 (75)	336 (70)	
III or IV	45 (25)	147 (30)	
Preoperative biliary drainage			0.179
No	107 (60)	257 (53)	
Yes	72 (40)	226 (47)	
Body mass index			0.006
<25	91 (51)	314 (65)	
≥25	88 (49)	169 (35)	
Tumor diameter			<0.001
<20 mm	66 (37)	101 (21)	
≥20 mm	113 (63)	382 (79)	
Lymph node metastases			0.001
No	111 (62)	155 (32)	
Yes	68 (38)	328 (68)	
Pathologist			0.767
Single	159 (89)	425 (88)	
Various	20 (11)	58 (12)	
Type of resection^a^			0.001
Pancreatoduodenectomy (PD)	102 (57)	362 (75)	
Pylorus-reserving PD	77 (43)	121 (25)	
Lymphadenectomy			0.001
Standard	156 (87)	328 (68)	
Radical	23 (13)	155 (32)	

^a^Including total pancreatectomy with resection of the distal stomach (classified as PD) and without (pylorus-preserving PD)

^b^Chi-square test; numbers in parentheses are percentages

**Table 4:** Multivariate analysis of predictive factors for higher lymph node counts (≥12)

Variable	Odds ratio	95 % confidence interval	*P*
BMI (≥25)	0.831	0.431–1.612	0.582
Pylorus-preserving resection (yes)	0.950	0.467–1.930	0.887
Diameter (≥20 mm)	2.547	1.225–5.329	0.013
Lymph node metastases (yes)	2.642	1.378–5.061	0.004
Lymphadenectomy (radical)	5.566	2.041–15.148	0.001

### Lymph node count and survival in patients with malignancies

**Fig. 1:**
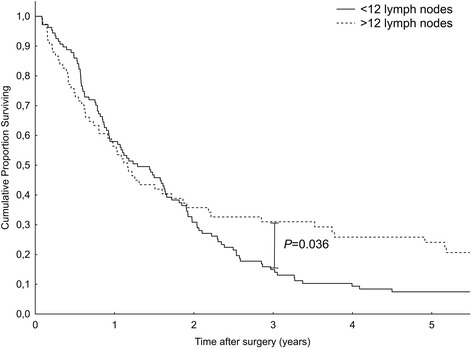
Kaplan-Meier survival curves for pancreatic cancer according to the number of evaluated lymph nodes. Patients with 12 or more lymph nodes removed had a significantly better long-term survival than those with 11 or fewer nodes (*P* = 0.036, log-rank test)

**Fig. 2:**
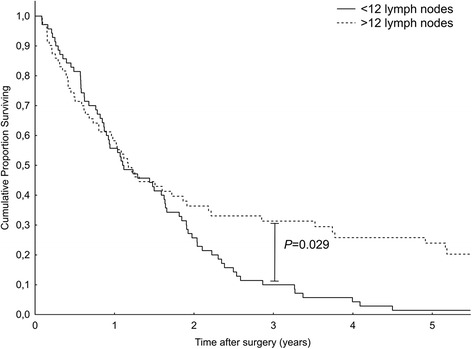
Prognostic effects of lymph node counts in patients with pancreatic cancer subject to standard lymphadenectomy. Higher number of resected lymph nodes (>12) was associated with improved survival (*P* = 0.029, log-rank test)

A group of 247 patients was alive after a median follow-up of 94 months (range 24–295 months, final follow-up December 2015). The overall median survival for patients with pancreatic, ampullary, and common bile duct cancers were 15 months (95 % CI 10.6–21.2), 52 months (95 % CI 34.9–68.9), and 18 months (95 % CI 10.6–25.4). A subgroup analysis carried out for individual cancer sites demonstrated that removing at least 12 lymph nodes is associated with better 3- and 5-year survival rates among patients with pancreatic cancer (Fig. 1). The survival benefit of higher nodal counts was maintained also in patients subject to standard lymphadenectomy (Fig. 2). No such effects were found for other malignancies. A subgroup analysis was also carried out for the effects of lymphadenectomy on patients’ survival, but the extent of lymph node dissection (standard vs radical) did not influence prognosis or perioperative complications.

## Discussion

Appropriate evaluation of lymph nodes in patients with solid tumors has obvious implications for more accurate staging. This study has demonstrated that the adequate lymph node yield with standard pathologic processing of pancreatoduodenectomy specimens in patients with suspected periampullary malignancy is influenced by only three factors, i.e., tumor diameter, metastases to lymph nodes and extent of lymphadenectomy. Moreover, evaluation of 12 or more nodes was associated with survival benefit in patients with pancreatic cancer.

The amount of lymphatic tissue and numbers of lymph nodes in the upper abdomen vary among individuals [19]. However, the yield of lymph nodes in all surgical specimens, including pancreatoduodenectomy, is mostly influenced by three main groups of variables, i.e., those related to the patient and underlying pathology, to surgical intervention, and to pathologic assessment of the specimen. Although tissue processing and thoroughness of the pathologic examination are the key factors for identifying lymph nodes in surgical specimens, there is no general agreement regarding the appropriate pathological evaluation of pancreatoduodenectomy specimens, leading to ambiguities in defining R1 resections and marked variability in counts of lymph nodes identified in the peripancreatic tissue [20, 21]. The average number of lymph nodes identified in such specimens with standard techniques of pathology sampling is five to seven [20]. Rarely, the number reaches 15 to 29 in some studies examining anatomic distribution of peripancreatic lymph nodes or those applying meticulous processing of the specimen [12, 22, 23]. The use of a standardized protocol for harvesting lymph nodes in our department and the fact that almost 70 % of the specimens were examined by a single pathologist minimize the risk that pathologist-related variability could bias results of the current study and give the opportunity to evaluate the influence of other factors on nodal yield.

There are very few studies reporting variables affecting the number of lymph nodes dissected in pancreatic surgery. Govindarajan et al. in a population of 2111 patients subject to pancreatoduodenectomy or total pancreatectomy for pancreatic head cancer from 1998 through 2003 and identified from the Surveillance, Epidemiology and End Results (SEER) registry found that younger age, female sex, tumor diameter >2 cm, and node-positive status increased the overall nodal count by 10 to 18 % [3]. Another analysis of the same database, but covering the period from 1993 through 2003, demonstrated that the likelihood of removing ten or more lymph nodes among 5465 pancreatoduodenectomies for periampullary carcinomas was higher in females, tumor diameter ≥2 cm, pancreatic head cancers, and metastases to regional lymph nodes [1]. However, the use of SEER data carries several disadvantages, including the unavailability of some variables potentially affecting lymph node counts such as BMI or the extent of lymphadenectomy. Another important issue that has not been addressed before is variability in lymph node counts among different surgeons and pathologists. In contrast with both previous studies, our database provides much more detailed information necessary to better characterize potential predictors of nodal yield.

The association between the lymph node yield and node positivity, as found in this study, is somewhat controversial. Besides the SEER studies, two previous reports suggested that in patients with pancreatic cancer subject to various pancreatic resections there was a significant difference in the total lymph node count in cases with or without nodal metastases of 19 vs 13 (*P* = 0.02) [5] and 15 vs 10 (*P* < 0.001) [24]. However, some other studies failed to confirm such a relationship [6, 25–27]. These discrepancies may derive from two potential aspects. First, the presence of enlarged, metastatic nodes may force the operating surgeon to a more extended dissection, and second, metastatic lymph nodes are usually larger and thus easier to identify by the pathologist. The proportion of patients subject to radical lymphadenectomy in our study was not influenced by the presence of metastatic lymph nodes, but the overall median number of nodes was significantly higher in this group (18 vs 15, *P* < 0.001). As a subsequent analysis in individual nodal stations revealed that the median count among subjects with metastatic nodes was higher only for pancreaticoduodenal stations (9 vs 7, *P* = 0.040), we may assume that lymph node metastases did not affect the extent of surgery.

As reasonably expected, our study revealed that performing radical lymph node dissection, including para-aortic nodes, is the most significant factor and the only surgeon-dependent one to achieve the recommended nodal yield. Although none of the prospective randomized clinical trials on the extent of lymphadenectomy in periampullary malignancies analyzed variables that potentially affect nodal yield, median numbers of lymph nodes dissected in these studies during standard pancreatoduodenectomy was 13 to 17 and for extended 20 to 36 [23, 25, 28, 29]. The idea of radical dissection is further supported by data accumulated over recent years suggesting that the incidence of lymph node metastasis to para-aortic nodal stations in periampullary malignancies is relatively high and an appropriate degree of lymphadenectomy is necessary to achieve an R0 resection [30–35]. Although a recent meta-analysis of sixteen studies comprising 1909 patients comparing outcomes of standard and extended pancreatoduodenectomy showed similar perioperative morbidity and mortality rates, it also emphasized no improved survival after the latter procedure (hazard ratio 0.77, *P* = 0.100) [36]. Therefore, the only benefit of the latter procedure seems to be associated with more accurate staging of nodal disease even if some controversies still exist about station 16b1 considered as one of the major lymphatic drainage routes for pancreatic head cancer [18].

Removal of the recommended number of lymph nodes was not associated with any clear survival benefit in the whole population of patients with periampullary malignancies. However, if the tumors were analyzed separately, pancreatic cancer demonstrated better survival among patients with ≥12 lymph nodes resected regardless the extent of lymphadenectomy. This is similar to some observations using cutoff values of 10 or 12 lymph nodes; however, the relationship between node counts and survival is not clear as previous studies on periampullary malignancies reported conflicting results [1, 3–6, 37].

The limitations of the present study are related to its retrospective design and potential bias resulting from such analyzes. In particular, we were unable to account for the premises for performing a more extensive lymph node dissection, such as finding suspicious nodes intraoperatively or decisions made a priori, even though data analysis showed no such correlation. Nevertheless, the lack of major changes observed over time in the absolute number of lymph nodes harvested and the proportion of patients with ≥12 nodes support the assumption that the surgical technique remained unchanged over the study period.

## Conclusions

In conclusion, this study demonstrated that only few factors were associated with the likelihood of removing at least 12 lymph nodes in surgical specimens of patients subject to pancreatoduodenectomy for suspected periampullary malignancy, i.e., tumor diameter, lymph node metastases, and radical lymphadenectomy. However, the latter and the only modifiable factor offered no clear survival benefit in previous randomized clinical trials and potentially may increase postoperative morbidity. Therefore, appropriate pathology training should be considered to increase nodal yield rather than more aggressive lymphatic dissection.

## Abbreviations

AJCC: American Joint Committee on Cancer
ASA: American Society of Anesthesiologists
BMI: Body mass index
CI: Confidence interval
IQR: Interquartile range
JPS: Japan Pancreas Society
JSBS: Japanese Society of Biliary Surgery
OR: Odds ratio
PD: Pancreatoduodenectomy
SEER: Surveillance, Epidemiology, and End Results
TNM: Tumor, lymph nodes, distant metastases
